# The Proof of the Pudding Is in the Tasting: Data Are Needed to Test Models and Hypotheses Related to Compulsive Sexual Behaviors

**DOI:** 10.1007/s10508-018-1167-x

**Published:** 2018-02-20

**Authors:** Mateusz Gola, Marc N. Potenza

**Affiliations:** 1 Swartz Center for Computational Neuroscience, Institute for Neural Computations, University of California San Diego, 9500 Gilman Drive, San Diego, CA 92093-0559 USA; 2 Interdisciplinary Center for New Technologies, University of Nicolas Copernicus, Torun, Poland; 3 Departments of Psychiatry and Neurobiology, Child Study Center and the National Center on Addiction and Substance Abuse, Yale School of Medicine, New Haven, CT USA; 4 Connecticut Mental Health Center, New Haven, CT USA

Walton, Cantor, Bhullar, and Lykins (2017) recently reviewed the state of knowledge on problematic hypersexuality and presented a theoretical model of compulsive sexual behaviors (CSBs). Of note, their literature search was completed in September 2015 and multiple advances have been made since that time. Importantly, while multiple theoretical models and hypotheses have been forwarded over time regarding CSB and related behaviors, many models and hypotheses still await formal empirical evaluation. Nonetheless, recent studies have suggested future lines of investigation to formally test the models and hypotheses proposed. In this Letter, we focus on some of the questions raised by Walton et al. based on recent findings and indicate important unanswered questions which warrant research consideration to promote systematic progress.

## Unanswered Questions

*What is the prevalence of CSB?*

Walton et al., similar to other authors (Carnes, 1991), say that the estimated prevalence of CSB is between 2 and 6% of the general adult population. Unfortunately, definitions regarding what constitutes CSB remain debated, complicating the precise estimates of the prevalence of CSB. A similar situation existed for internet gaming disorder (IGD) where prevalence estimates ranged widely prior to the introduction of formal proposed criteria in the fifth edition of the *Diagnostic and Statistical Manual of Mental Disorders* (DSM-5; APA, 2013; Petry & O’Brien, 2013). Furthermore, no nationally representative data to date have been published to provide estimates of CSB, with existing data typically relying on convenience samples (Odlaug et al., 2013). It is very important to collect data from representative samples in order to understand the prevalence (and ideally impact) of CSB in the general population, and how it may differ between jurisdictions and across different groups (e.g., with respect to age, gender, culture). Such information may help us understand how specific factors (e.g., access to pornography, cultural values or norms, religious beliefs) may relate to specific types or forms of CSB.

A related question involves potential differences between clinical and subclinical populations. One example may relate to Walton et al.’s discussion of a role for religiosity in CSB. Two studies (Grubbs, Exline, Pargament, Hook, & Carlisle, 2015a; Grubbs, Volk, Exline, & Pargament, 2015b) provide support that religiosity and moral disapproval of pornography use may contribute to self-perceptions of porn addiction. On the other hand, Reid, Carpenter, and Hook (2016) found that religiosity was unrelated to self-reported measures of hypersexuality. Possible explanation for seeming discrepancies may involve methodological aspects (e.g., relating to how CSB is defined and assessed), differences in the populations studied, or other factors. With respect to the populations studies, Grubbs et al. focused on non-clinical (non-treatment-seeking) individuals while Reid et al. assessed subjects meeting criteria for hypersexual disorder (Kafka, 2010). In our recent study (Gola, Lewczuk, & Skorko, 2016a), we examined whether religiosity may contribute differently in these two populations in Poland. Using structural equation modeling, we examined relationships between amount of pornography use, negative health correlates of pornography use, religiosity, and treatment-seeking status for CSB. We collected data from 132 males seeking treatment for problematic pornography use, referred by clinical psychologists (and meeting criteria for HD), and 437 males using pornography on a regular basis but never seeking treatment. We found that religiosity was associated with self-perceived negative symptoms of pornography use in the non-treatment-seeking males but not in the treatment-seeking males. We also observed that while amount of pornography use did not statistically predict treatment-seeking status, severity of pornography-use-related negative symptoms did. These findings were observed despite similar levels of religiosity between the treatment-seeking and non-treatment-seeking populations (Gola et al., 2016a). Furthermore, findings may differ for women, as we recently observed that religiosity and amount of pornography use related to treatment-seeking for CSB among women (Lewczuk, Szmyd, Skorko, & Gola, 2017). These findings highlight the importance of studying CSB topics in a gender-informed fashion with additional considerations extending to cis- and transgendered populations and heterosexual, homosexual, bisexual, polyamorous, and other groups.

*What data are needed to inform conceptualizations of CSB?*

As described elsewhere (Kraus, Voon, & Potenza, 2016a), there is an increasing number of publications on CSB, reaching over 11,400 in 2015. Nonetheless, fundamental questions on the conceptualization of CSB remain unanswered (Potenza, Gola, Voon, Kor, & Kraus, 2017). It would be relevant to consider how the DSM and the *International Classification of Diseases* (ICD) operate with respect to definition and classification processes. In doing so, we think it is relevant to focus on gambling disorder (also known as pathological gambling) and how it was considered in DSM-IV and DSM-5 (as well as in ICD-10 and the forthcoming ICD-11). In DSM-IV, pathological gambling was categorized as an “Impulse-Control Disorder Not Elsewhere Classified.” In DSM-5, it was reclassified as a “Substance-Related and Addictive Disorder.” The rationale for this reclassification was based on existing data supporting similarities in multiple domains, including phenomenological, clinical, genetic, neurobiological, therapeutic, and cultural (Petry, 2006; Potenza, 2006), as well as differences in these domains with respect to competing models like obsessive–compulsive-spectrum classification (Potenza, 2009). A similar approach should be applied to CSB, which is currently being considered for inclusion as an impulse-control disorder in ICD-11 (Grant et al., 2014; Kraus et al., 2018). However, questions exist as to whether CSB is more similar to addictive disorders than the other impulse-control disorders (intermittent explosive disorder, kleptomania, and pyromania) proposed for ICD-11 (Potenza et al., 2017).

Among the domains that may suggest similarities between CSB and addictive disorders are neuroimaging studies, with several recent studies omitted by Walton et al. (2017). Initial studies often examined CSB with respect to models of addiction (reviewed in Gola, Wordecha, Marchewka, & Sescousse, 2016b; Kraus, Voon, & Potenza, 2016b). A prominent model—the incentive salience theory (Robinson & Berridge, 1993)—states that in individuals with addictions, cues associated with substances of abuse may acquire strong incentive values and evoke craving. Such reactions may relate to activations of brain regions implicated in reward processing, including the ventral striatum. Tasks assessing cue reactivity and reward processing may be modified to investigate the specificity of cues (e.g., monetary versus erotic) to specific groups (Sescousse, Barbalat, Domenech, & Dreher, 2013), and we have recently applied this task to study a clinical sample (Gola et al., 2017). We found that individuals seeking treatment for problematic pornography use and masturbation, when compared to matched (by age, gender, income, religiosity, amount of sexual contacts with partners, sexual arousability) healthy control subjects, showed increased ventral striatal reactivity for cues of erotic rewards, but not for associated rewards and not for monetary cues and rewards. This pattern of brain reactivity is in line with the incentive salience theory and suggests that a key feature of CSB may involve cue reactivity or craving induced by initially neutral cues associated with sexual activity and sexual stimuli. Additional data suggest that other brain circuits and mechanisms may be involved in CSB, and these may include anterior cingulate, hippocampus and amygdala (Banca et al., 2016; Klucken, Wehrum-Osinsky, Schweckendiek, Kruse, & Stark, 2016; Voon et al., 2014). Among these, we have hypothesized that the extended amygdala circuit that relates to high reactivity for threats and anxiety may be particularly clinically relevant (Gola, Miyakoshi, & Sescousse, 2015; Gola & Potenza, 2016) based on observation that some CSB individuals present with high levels of anxiety (Gola et al., 2017) and CSB symptoms may be reduced together with pharmacological reduction in anxiety (Gola & Potenza, 2016). However, these studies currently involve small samples and additional research is needed.

## Conclusion

In summary, we highlight the importance of empirical validation of models of CSB. Consensus is needed regarding the definition of CSBs and CSB disorder. If CSB disorder is included in ICD-11 as currently proposed, this could provide the foundation for systematic research in multiple domains. Well-designed and conducted longitudinal neuroscientific studies of CSB and non-CSB groups, including investigations allowing measurement of brain activity during actual sexual activity, could be very informative. We believe that such data may be used to test and refine existing models and permit the generation of new theoretical models developed in a data-driven fashion.
